# Noradrenergic dysfunction in Alzheimer's disease

**DOI:** 10.3389/fnins.2015.00220

**Published:** 2015-06-17

**Authors:** Mary Gannon, Pulin Che, Yunjia Chen, Kai Jiao, Erik D. Roberson, Qin Wang

**Affiliations:** ^1^Department of Cell, Developmental and Integrative Biology, University of Alabama at BirminghamBirmingham, AL, USA; ^2^Department of Genetics, University of Alabama at BirminghamBirmingham, AL, USA; ^3^Department of Neurology, University of Alabama at BirminghamBirmingham, AL, USA

**Keywords:** noradrenergic system, norepinephrine, adrenergic receptors, degeneration, dysfunction

## Abstract

The brain noradrenergic system supplies the neurotransmitter norepinephrine throughout the brain via widespread efferent projections, and plays a pivotal role in modulating cognitive activities in the cortex. Profound noradrenergic degeneration in Alzheimer's disease (AD) patients has been observed for decades, with recent research suggesting that the locus coeruleus (where noradrenergic neurons are mainly located) is a predominant site where AD-related pathology begins. Mounting evidence indicates that the loss of noradrenergic innervation greatly exacerbates AD pathogenesis and progression, although the precise roles of noradrenergic components in AD pathogenesis remain unclear. The aim of this review is to summarize current findings on noradrenergic dysfunction in AD, as well as to point out deficiencies in our knowledge where more research is needed.

## Introduction

Noradrenergic neurons are located primarily in the locus coeruleus. Via wide spread efferent projections, these neurons provide norepinephrine throughout the brain (Swanson and Hartman, 1975). Along with dopamine and epinephrine, norepinephrine is a catecholamine that is synthesized through a series of enzymatic steps that begins with the amino acid tyrosine. Tyrosine is first converted to levodopa (L-DOPA) by the enzyme tyrosine hydroxylase, which is the rate-limiting step. Pyridoxal phosphate and DOPA decarboxylase then convert L-DOPA to dopamine, which is subsequently converted to norepinephrine by dopamine beta hydroxylase (DβH).

After synthesis, norepinephrine is released by presynaptic noradrenergic terminals, and can bind to receptors, be degraded by enzymes such as catechol-O-methyltransferases (COMT) or monoamine oxidases (MAO) (Golan et al., 2011), or undergo reuptake into the presynaptic neuron. Norepinephrine elicits its effects through three classes of adrenergic receptors, the α_1_, the α_2_, and the β adrenergic receptors, each of which consists of three subtypes (Hein, 2006). While all of the receptors are located at postsynaptic neurons, the α_2_ receptors are uniquely also located presynaptically where they function as autoreceptors to inhibit the further release of norepinephrine (Knaus et al., 2007). The amount of norepinephrine available to bind to receptors is largely dependent on either degradation or reuptake of norepinephrine to the presynaptic terminals. Norepinephrine reuptake is mediated by the norepinephrine transporter, a Na/K pump that returns norepinephrine to the presynaptic terminal.

Changes in the noradrenergic system have long been observed in Alzheimer's disease (AD). Pathological changes of noradrenergic neurons in the locus coeruleus, including the accumulation of tau protein (Braak and Del Tredici, 2012), begin early in the progression of AD. Later in the disease, profound loss of the noradrenergic neurons is observed, along with compensatory changes such as modifications of neuronal anatomy, neurotransmitter, and noradrenergic receptors. Given the well-established functions of the noradrenergic system in cognition, neuroinflammation, and metabolism, it could be predicted that noradrenergic dysfunction in AD would promote disease progression. Indeed, evidence from both human patients and animal models indicates that loss of noradrenergic innervation greatly exacerbates AD pathogenesis and progression (Marien et al., 2004; Heneka et al., 2006; Grudzien et al., 2007; Kalinin et al., 2007; Pugh et al., 2007). There is currently no effective treatment or cure for AD. Understanding the changes that occur in the noradrenergic system in AD patients could present a unique opportunity to inspire new avenues of treatment. The purpose of this review is to describe what is currently known about noradrenergic dysfunction in AD and its potential contribution to AD pathology and progression, as well as to point out deficiencies in our knowledge where more research is needed.

## The role of the noradrenergic system in cognition

The role of the noradrenergic system on cognition has been reviewed extensively elsewhere (Chamberlain and Robbins, 2013). We provide a brief overview of the literature here as an introduction to the role of the noradrenergic system in AD, but we are aware that we have not mentioned all relevant papers to this topic.

### Importance of norepinephrine

Examining the effects of norepinephrine depletion has revealed the importance of norepinephrine in cognitive tasks. Depletion of norepinephrine in animal and cellular models has been achieved in three different ways. First, norepinephrine depletion has been accomplished through degeneration of noradrenergic neurons with DSP-4, a neurotoxin selective for noradrenergic neurons. While this most completely depletes norepinephrine, it also causes structural changes in the brain and therefore the changes may not necessarily be completely attributed to the neurotransmitter loss. Another method of norepinephrine depletion is the use of reserpine to block the vesicular monoamine transporter (VMAT), which transports monoamines into vesicles in the presynaptic terminal for synaptic release. It is important to note that reserpine is not specific to norepinephrine and works on all monoamines, including dopamine and serotonin. A final method of norepinephrine depletion is through the knockout of DβH (the enzyme converting dopamine to norepinephrine) to look exclusively at the effects of norepinephrine. Despite of the potential limitation associated with each method, studies with these independent methods have demonstrated that loss of norepinephrine leads to cognitive impairment in various aspects of cognition.

Depletion of norepinephrine in rats by DSP-4 leads to deficits in working memory (Sontag et al., 2008). This deficit has also been shown to be exacerbated when in combination with the depletion of other neurotransmitters, either dopamine (Pérez et al., 2009) or acetylcholine (Ohno et al., 1997). Treating monkeys with reserpine also induces a working memory deficit (Cai et al., 1993). Deficits in spatial memory, as measured by the Morris water maze, have also been reported following norepinephrine depletion with DSP-4, and this effect is especially significant in stressed and aged rats (Sirviö et al., 1991; Lapiz et al., 2001). In addition, DSP treatment impairs visual memory (Rajabi et al., 2012), olfactory memory (Guan et al., 1993) and avoidance learning, and administration of epinephrine rescues the avoidance learning defect caused by norepinephrine depletion (Bennett et al., 1990). DβH deficient mice also show impaired active-avoidance learning (Thomas and Palmiter, 1997) and social memory (Marino et al., 2005). Together, these studies strongly support a critical role of norepinephrine in cognitive functions.

An alternate method of studying the effect of norepinephrine on cognition is by increasing the availability of this neurotransmitter. The selective norepinephrine reuptake inhibitor atomoxetine increases the amount of norepinephrine available in the synapse by preventing its reuptake by the presynaptic terminals. Increasing norepinephrine improves spatial learning and memory of rats in both the novel object task and the radial arm maze (Tzavara et al., 2006). Additionally, improvements in working memory (Gamo et al., 2010) and attention (Tzavara et al., 2006; Jentsch et al., 2009) have been seen after atomoxetine treatment. These results are consistent with the findings from the depletion studies; while a decrease in norepinephrine induces cognitive deficits, increasing norepinephrine can improve cognitive function.

### Differential roles of the adrenergic receptors

All nine adrenergic receptors are members of the G-protein coupled receptor family. The α_1_ receptors all signal through the G_q_ signaling pathway and cause increases in both IP_3_ and calcium upon activation (Chen and Minneman, 2005; Perez, 2007). The β receptors all signal through the G_*s*_ signaling pathway and activation of these receptors leads to increased cAMP. The β_2_ receptor is also able to signal through the G_i_ signaling pathway to modulate cAMP and lead to ERK activation (Hall, 2004; Xiang, 2011). The α_2_ receptors signal through the G_i/o_ signaling pathway to inhibit both calcium and cAMP (Knaus et al., 2007). The α_2_ receptors can serve as autoreceptors on presynaptic noradrenergic terminals, inhibiting the further release of norepinephrine. The known functions of different adrenergic receptors in cognition are summarized in Table 1.

**Table 1:** **The known functions of adrenergic receptors in cognition**.

**Receptor**	**Subtype**	**Function in cognition**	**Localization of function**
α_1_	α_1A_	Improve spatial learning (Doze et al., 2011)	Hippocampus
	α_1B_	Improve fear learning (Nalepa et al., 2013)	Amygdala
	α_1D_	Improve working memory and attention (Mishima et al., 2004)	Prefrontal cortex
α_2_	α_2A_	Impair spatial and fear learning (Gamache et al., 2012; Warner and Drugan, 2012; Zoladz et al., 2013; Torkaman-Boutorabi et al., 2014)	Hippocampus
		Improve working memory (Arnsten and Goldman-Rakic, 1985, 1987; Arnsten et al., 1988)	Prefrontal cortex
	α_2B_	Unknown	–
	α_2C_	Unknown	–
β	β_1_	Improve auditory fear memory (Qu et al., 2008)	Administration into amygdala
		Impair spatial reference memory	Hippocampus
	β_2_	Improve memory retrieval (Introini-Collison et al., 1991)	Administration into amygdala
		Improves fear memory (Zhou et al., 2013)	Administration into prefrontal cortex
		Improve auditory fear memory (Qu et al., 2008)	Administration into amygdala
	β_3_	unknown	–

The α_1_ receptor has been investigated in both fear conditioning and spatial learning. Overall, inhibition of the α_1_ receptor impairs learning while activation of the receptor improves it. In fear learning, antagonism of the α_1_ receptor leads to poorer performance in these tasks. Prazosin, an α_1_antagonist, has been shown to cause both a diminished fear response in an olfactory fear conditioning paradigm (Do Monte et al., 2013) and in an olfactory recall task (Veyrac et al., 2007). In spatial learning, prazosin induces impairment (Torkaman-Boutorabi et al., 2014) and also exacerbates a deficit induced by scopolamine, a muscarinic cholinergic antagonist (Puumala et al., 1998). Consistent with the antagonist data, treatment with α_1_agonists has been shown to improve spatial memory in the Morris water maze (Puumala et al., 1996, 1998). It is worth noting that prazosin may have an inhibitory effect on overall locomotion in the Morris water maze (Levcik et al., 2013), which would confound the spatial memory test.

Genetic studies have revealed subtype specific roles for the α_1_ receptors with the α_1A_ knockout mice showing the most dramatic cognitive deficits. The α_1A_ knockout mice display poor spatial learning and memory in both the Barnes maze and Morris water maze (Doze et al., 2011). α_1B_ knockout mice show increased short-term-latency and a decline in long-term-latency in passive avoidance tests, suggesting that the α_1B_ receptor plays a role in fear-motivated exploratory behavior (Nalepa et al., 2013). While α_1D_ knockout mice show no difference in spatial or emotional learning when compared to WT mice (Sadalge et al., 2003), these mice have a deficit in working memory and attention (Mishima et al., 2004).

The α_2_ receptors are involved in several different paradigms of learning. Antagonism of α_2_ receptors has been shown to benefit memory and cognition in a number of settings. Yohimbine, an α_2_ antagonist, increases fear conditioning (Gazarini et al., 2013) and improves spatial cognitive performance in the Morris water maze (Torkaman-Boutorabi et al., 2014). Additionally, yohimbine effectively improves both accuracy and response latencies in the habituated animal (Brown et al., 2012). Another α_2_ antagonist, dexefaroxan, improves odor learning (Veyrac et al., 2007), spatial and visual memory, and passive avoidance (Chopin et al., 2002). The α_2_ antagonist atipamezole enhances spatial learning in aged rats (Haapalinna et al., 2000). α_2_ receptor blockade may also potentiate cholinergic activity in the formation of a long-term memory trace; the α_2_ antagonists yohimbine, idazoxan, and P86 7480 are all able to enhance passive avoidance learning when co-administered with heptylphysostigmine, a cholinesterase inhibitor (Camacho et al., 1996).

Pharmacological activation of the α_2_ receptors also leads to cognitive changes. In a model of the effect of stress on spatial learning, clonidine exacerbates the learning deficits produced from the stress of cold water in the Morris water maze (Warner and Drugan, 2012). Clonidine also weakens reconsolidation of fear memories, and has therefore recently been looked at as a potential treatment for PTSD (Gamache et al., 2012; Zoladz et al., 2013). While activation of the α_2A_ receptors induces deficits in spatial and fear learning, it actually has been shown to enhance working memory. Clonidine and guanfacine can improve working memory in aged monkeys with documented memory impairment (Arnsten and Goldman-Rakic, 1985, 1987; Arnsten et al., 1988). It is known that working memory is mainly localized to the prefrontal cortex (Wang et al., 2013c) whereas spatial and fear memory involve the hippocampus (Bird and Burgess, 2008). The difference in clonidine's effects on these different cognitive paradigms is likely due to diverse functions of α_2_ receptors in regulating neurotransmission in different brain regions. α_2_ receptor stimulation enhances firing of prefrontal cortical neurons through inhibition of HCN channels (Wang et al., 2007). On the other hand, α_2_ receptor activation blocks hippocampal long-term plasticity (McMahon and Wang, unpublished findings).

There is limited information on the role of the α_2_ receptors in cognition from genetically modified mice. α_2A_(the most prevalent subtype in the CNS) knockout mice show impairment in working memory (Franowicz et al., 2002). Changes in spatial or fear learning in these mice remain uninvestigated.

Much of our knowledge of the role of the β adrenergic receptors in cognition comes from pharmacological studies. Pharmacological activation of β receptors using the agonist isoproterenol has been found to enhance long term potentiation and memory consolidation in the hippocampus (Gelinas et al., 2008). On the other hand, the β antagonist propranolol inhibits memory retrieval in extinction tasks (Ouyang and Thomas, 2005) and in a fear conditioning paradigm (Taherian et al., 2014). Propranolol also inhibits taste memory consolidation (Ruetti et al., 2014) and impairs scent recall (Veyrac et al., 2007). Additionally, administration of propranolol with scopolamine, a muscarinic cholinergic antagonist, or with p-chlorophenylalanine, a serotonergic antagonist, synergistically impairs rat spatial learning in the Morris water maze (Decker et al., 1990; Saber and Cain, 2003; Kenton et al., 2008). Together, these studies indicate a positive role of β adrenergic receptors in memory consolidation in general.

Effects of β_1_ and β_2_ subtype-selective ligands on cognition have also been investigated. Infusion of the β_2_ agonist clenbuterol into the amygdala facilitates memory retention in an inhibitory avoidance task (Introini-Collison et al., 1991), and infusion of clenbuterol into the prefrontal cortex enhances trace fear memory (Zhou et al., 2013). On the other hand, infusion of β_1_ or β_2_ antagonists into the amygdala causes a deficit in auditory fear memory (Qu et al., 2008). However, xamoterol, a partial β_1_ receptor agonist, impairs the retrieval of hippocampus-dependent spatial reference memory in rats (Schutsky et al., 2011), suggesting that different subtypes of β receptors may play different roles in regulating cognition in different brain regions.

## Other functions of the noradrenergic system potentially related to AD

It is well-established that the brains of AD patients have significant neuroinflammation (Hensley, 2010). Both astrocytes and microglia have adrenergic receptors present (Salm and Mccarthy, 1989; Shao and Sutin, 1992; Sutin and Shao, 1992; Tanaka et al., 2002), and norepinephrine regulates inflammatory processes through these receptors. Loss of neurons in the locus coeruleus and the subsequent loss of norepinephrine result in an increase in inflammation in animal models (Heneka et al., 2006, 2010; Jardanhazi-Kurutz et al., 2011). Furthermore, stimulating microglia with norepinephrine led to increased phagocytosis of amyloid-β (Aβ) by microglia (Heneka et al., 2010). Taken together, this indicates that alterations of the noradrenergic system at least partially contribute to the neuroinflammation seen in AD and the pathogenic consequences of that inflammation.

Another symptom of AD is aberrant glucose metabolism (Schubert, 2005), which can be seen in the increased levels of oxidative stress in AD patients that occurs before the appearance of Aβ plaques (Perry et al., 2002). α_1_ receptors on astrocytes regulate glucose uptake. Additionally, activation of the α_2_ receptors on astrocytes leads to an increase of glycogenesis, whereas activation of the β-receptors promotes glycogenolysis (O'donnell et al., 2012). This indicates that the adrenergic receptors play a large role in the balance of glycogen in the brain and, therefore, could potentially be targeted to improve the aberrant glucose metabolism seen in AD.

## Noradrenergic changes in AD

There is considerable evidence showing that the normal anatomy and functions of the noradrenergic system are altered in AD. We will consider these data at the level of neurons, neurotransmitters, and receptors.

### Changes in locus coeruleus neurons

At an anatomical level, it has been well-established that AD patients show a loss of noradrenergic neurons in the locus coeruleus (Mann et al., 1980; Iversen et al., 1983; Forstl et al., 1994; Matthews et al., 2002; Zarow et al., 2003). Though there is an overall loss in neurons, there is actually an increase in both dendritic and axonal sprouting from the remaining noradrenergic neurons in the locus coeruleus. In patients with dementia, the dendritic sprouting from the locus coeruleus to the prefrontal cortex after neuronal loss keeps the levels of connections stable or even slightly increased, as measured by presynaptic α_2_ and postsynaptic α_1_ receptor density (Szot et al., 2007). Axonal sprouting of the remaining locus coeruleus neurons to the hippocampus also occurs in patients with noradrenergic neurodegeneration (Szot et al., 2006). Recently, technological advances have allowed for the detection of locus coeruleus neuron loss in living patients. Using high-resolution fast spin-echo T1-weighted imaging, Takahashi et al. (Takahashi et al., 2015) were able to detect a decrease in locus coeruleus density in AD patients when compared to controls. This advance is exciting as it could allow for the early detection of locus coeruleus neuron loss, which occurs at early stages of AD.

While the noradrenergic changes are commonly seen in AD patients, few commonly used AD mouse models actually show the locus coeruleus degeneration. A Down syndrome mouse model that includes a triplication of the APP gene shows degeneration of the locus coeruleus neurons with aging, which has been linked to the overexpression of APP (Salehi et al., 2009; Lockrow et al., 2011). Tg2576 mice also display some degree of noradrenergic changes, including neurodegeneration in the locus coeruleus (Liu et al., 2008; Guerin et al., 2009; Eimer and Vassar, 2013). However, many other AD rodent models do not have neurodegeneration, which is a limitation of these models.

### Changes in norepinephrine levels

Despite the loss of the noradrenergic neurons, there have been conflicting reports on levels of norepinephrine in the brain, with some studies showing a decrease in norepinephrine while others showing that norepinephrine levels in AD patients remain constant, or even elevated. Using either HPLC (Martignoni et al., 1992; Nazarali and Reynolds, 1992; Matthews et al., 2002) or a fluorophore based method (Reinikainen et al., 1988), several groups have found a decrease in norepinephrine concentration in various brain regions, with the loss proportional to the level of cognitive deficit (Matthews et al., 2002). Norepinephrine turnover is increased in the remaining locus coeruleus neurons. The concentration of 3-methoxy-4-hydroxyphenylglycol (MHPG), a norepinephrine metabolite, has been used to indicate the norepinephrine turnover rate. AD patients have a higher ratio of the norepinephrine metabolite to norepinephrine (Palmer et al., 1987; Hoogendijk et al., 1999). This increased metabolism of norepinephrine is a possible cause of the decreased norepinephrine levels. On the other hand, a number of studies have shown no change in norepinephrine levels in different brain regions or cerebrospinal fluid (CSF) in both sporadic and familial AD cases (Sparks et al., 1988; Herregodts et al., 1989; Tohgi et al., 1992). In addition, increased norepinephrine levels were reported, which correlated with decreased cognitive function (Tohgi et al., 1992) and aging (Elrod et al., 1997) in AD patients.

There is evidence of compensatory changes that can account for the loss in noradrenergic neurons, yet stable or increased norepinephrine levels. One finding has been that norepinephrine transporter sites are decreased in AD patients. In autoradiograph studies (Gulyas et al., 2010) and radioligand binding studies of post-mortem tissues (Tejani-Butt et al., 1993), the norepinephrine transporter sites were significantly reduced in AD brains compared to normal control samples. As norepinephrine transporter is responsible for norepinephrine reuptake to the presynaptic neurons, a decrease in norepinephrine transporter sites would increase the amount of norepinephrine in the synapse. Another possible mechanism for norepinephrine increase is an increase in the enzymes responsible for the production of norepinephrine. Tyrosine hydroxylase, which catalyzes the rate-limiting step in the production of norepinephrine, is increased in the brain of AD patients (Iversen et al., 1983; Szot et al., 2006, 2007). The enzyme DβH is also increased in serum, CSF, and peripheral blood lymphocytes in AD cases (Miyata et al., 1984; Giubilei et al., 2004).

With researchers reporting both increases and decreases in norepinephrine levels in AD patients, it is necessary to look deeper into the discrepancy. The differences are likely due to the stage of the disease and the brain's ability to compensate for the neuronal loss. One possible theory is that in the early stages of locus coeruleus neuron loss, the remaining neurons undergo compensatory mechanisms to maintain the norepinephrine level. However, as the disease progresses and more neurons are lost, it may become impossible for the remaining neurons to totally compensate. It is also possible that the brain over-compensates for the neuronal loss, which can account for the increased norepinephrine levels reported in the later stages of the disease. Eventually, however, if the disease progresses enough, the loss of locus coeruleus neurons will be too great to overcome and norepinephrine levels will decline. This hypothesis could be tested by using the new high-resolution fast spin-echo T1-weighted imaging to measure locus coeruleus density and correlating it to the norepinephrine levels in the CSF of AD patients (Takahashi et al., 2015).

### Changes in noradrenergic receptors

In addition to the anatomical and neurotransmitter changes, there are also changes to the adrenergic receptors of AD patients. Changes in the receptors in postmortem brain tissues have been studied using both binding assays and mRNA quantification. These studies indicate several changes in the AD brain, which would lead to altered functional outcome in response to norepinephrine.

α_1_ receptor density has been examined using non-subtype selective radioligands. Decreases in α_1_ receptor density have been reported in the prefrontal cortex (Kalaria, 1989), hippocampus, and cerebellum (Shimohama et al., 1986). However, when examining subregions, Szot et. al. found stable to slightly increased α_1_ binding sites in layers I/II of the prefrontal cortex (Szot et al., 2007) and in the molecular layer of the dentate gyrus of the hippocampus (Szot et al., 2006). Additionally, changes in specific α_1_ receptor subtypes have been examined, with a reduction of α_1C_ mRNA in layers II/III of the prefrontal cortex and a significant decrease in α_1D_ mRNA in the hippocampus (Szot et al., 2007).

The density of α_2_ receptors has also been studied by the use of binding assays. Increased α_2_ receptor density has been found in cortical membranes (Ruiz et al., 1993) and the dentate gyrus granule cell layer (Szot et al., 2006) of post-mortem AD patient brains and in brains of patients with the AD related disease, dementia with Lewy bodies (Leverenz et al., 2001; Szot et al., 2006). The levels of α_2_ receptors in brain microvessels, which are innervated by locus coeruleus noradrenergic neurons (Kalaria et al., 1989b), are also increased in AD patients (Kalaria et al., 1989a). However, there are also reports of no significant change (Shimohama et al., 1986; Szot et al., 2007) in α_2_ receptor density in the cortex or a decrease in α_2_ density in the hippocampus (Pascual et al., 1992) or nucleus basalis of Meynert (Shimohama et al., 1986) of AD patients. One thing that could be contributing to the discrepancy of the above reports is that studying α_2_ receptor density is a more complicated endeavor due to their unique presence on the presynaptic neuron. This is especially true considering the neuronal loss that occurs in the locus coeruleus of AD patients, and the fact that α_2_ receptors are located on those neurons. Therefore, even if there is actually an increased density of α_2_ receptors on the remaining locus coeruleus neurons or at the projection sites, locus coeruleus neuron loss could mask that increase due to the lost receptors on the degenerated neurons. Indeed, Szot et al. (2007) found that the loss of locus coeruleus neurons was greater than the α_2_ receptor loss in the cortex area with locus coeruleus neuron projections. This probably indicates an increased number of receptors per neuron in AD cases.

Changes in α_2_ receptor subtypes have also been examined in AD patients. By measuring the mRNA at different projection sites to isolate just the postsynaptic receptors, Szot et al. (2007) found decreased levels of α_2A_ mRNA in the prefrontal cortex, though the reduction was limited to layer II. In the hippocampus, levels of α_2A_ mRNA were unchanged, but there was a significant decrease in α_2C_ mRNA. Szot et al. (2006) also looked at presynaptic receptors in post mortem AD brains by measuring receptor mRNA from the locus coeruleus and found an overall reduction in α_2A_mRNA in AD patients without change in the number of receptors per cell. While examination of mRNA levels is useful to determine changes in different receptor subtypes, it may not reflect actual changes in receptor density. Therefore, these data should be interpreted with the results from the binding assays.

Finally, β receptor density in AD patient brains has been studied, though there is considerably less information on the β receptor changes. In both AD patients and controls, the β_1_ receptor density is higher in the cortex than the hippocampus, but there is no difference between the two groups (Lemmer et al., 1993). However, a decrease in β_2_ receptor density occurs in the microvessels of AD patients (Kalaria et al., 1989a).

Pathological changes of the noradrenergic system in AD are dynamic as the disease progresses. Most current studies look at one snapshot of the pathological process, which likely give discrepant results. Systematic examinations of various noradrenergic components at different disease stages and correlations of these changes with other AD-related pathological and cognitive deficits are necessary in order to fully comprehend noradrenergic dysfunction in AD.

## Potential involvement of noradrenergic dysregulation in AD pathogenesis and progression

### The role of norepinephrine

The first evidence for a functional role of noradrenergic dysregulation in AD comes from animal models in which the norepinephrine levels are manipulated. Treatment of AD transgenic mice with DSP-4, a neurotoxin which selectively ablates noradrenergic neurons, increases Aβ deposition (Heneka et al., 2006; Kalinin et al., 2007; Jardanhazi-Kurutz et al., 2010), impairs spatial memory (Jardanhazi-Kurutz et al., 2010) and alters α_1_, α_2_, and β_1_ receptor binding sites and mRNA expression (Jardanhazi-Kurutz et al., 2011). DSP-4 treatment also increases the levels of accumulated hyperphosphorylated tau in cortices of female APP-SL mice (Oikawa et al., 2010). APP/PS1 mice crossed with DβH^−/−^ mice, which are unable to synthesize norepinephrine, have compromised LTP and maze performance, which is worse than in either of the single mutants alone (APP/PS1 or DβH^−/−^) (Hammerschmidt et al., 2013). APP/PS1 mice crossed with Ear2^−/−^ mice, which show marked locus coeruleus neuron loss, have exacerbated LTP and memory deficits, but do not have plaque deposition different from APP/PS1 mice (Kummer et al., 2014). This indicates that the norepinephrine loss contributes to the cognitive dysfunction of AD, even in the absence of the hallmark pathogenic Aβ plaques. Taken together, this evidence suggests that loss of noradrenergic input to the cerebral cortex exacerbates AD-related pathological and behavioral deficits.

*In vivo* tests also suggest that norepinephrine supplementation could be beneficial in AD. Norepinephrine levels and microglial function can be rescued by peripherally administering the norepinephrine precursor, L-DOPS, in DSP-4 lesioned AD mice expressing mutant APP (APP/PS1 or APPV717I). Additionally, these same AD model mice have improved Aβ clearance, decreased Aβ plaques, and improved spatial memory after L-DOPS administration (Heneka et al., 2010; Hammerschmidt et al., 2013). Co-administering L-DOPS along with atomoxetine, a norepinephrine reuptake inhibitor, in 5xFAD transgenic mice improves learning in the Morris water maze test (Kalinin et al., 2012).

In addition to animal studies, human association studies support the role of norepinephrine loss in AD. In AD patients, the extent of noradrenergic degeneration correlates with both the degree of pathological changes (including amyloid plaques and neurofibrillary tangles) and the severity of cognitive deficits (Bondareff et al., 1987; Zarow et al., 2003). Additionally, there is a link between a low-activity polymorphism in the DβH gene and AD in a Caucasian population (Combarros et al., 2010). As DβH is required for the production of norepinephrine, lower activity of this enzyme leads to decreased norepinephrine. However, this polymorphism may be population-specific, as a study by Komatsu et al. (2014) looked at that same polymorphism along with another DβH polymorphism and did not find any link between either of the two different DβH polymorphisms and AD in a Japanese population.

### Potential involvement of the noradrenergic receptors

The potential role of α_1_ receptors in modulating AD-related pathological and behavioral changes has been studied using prazosin, an α_1_ receptor antagonist. Prazosin reduces the generation of Aβ in N2a cells and ameliorates memory loss in APP23 transgenic mice (Katsouri et al., 2013). However, since prazosin is not a subtype-selective antagonist, this study only suggests the involvement of the α_1_ receptor as a whole, and not the roles of the individual receptor subtypes. Further studies are needed to determine the specific subtypes of α_1_ receptors involved in the protective effect by prazosin.

Our recent studies have revealed the involvement of the endogenous α_2A_ subtype receptor in AD pathogenesis (Chen et al., 2014). Stimulation of the α_2A_ receptor significantly enhances, while genetic or pharmacological blockade of this receptor reduces, Aβ generation and Aβ-related neuropathology. Activation of α_2A_ receptor signaling disrupts endogenous APP interaction with sorting-related receptor with A repeat (SorLA), and consequently promotes amyloidogenic processing of APP in endosomes. We therefore provided the first evidence that SorLA-dependent APP sorting can be targeted by extracellular signaling to modulate amyloidogenesis. This is particularly important because it suggests a method of reducing Aβ production without targeting the activity of the secretases responsible for its generation. Significantly, there have been reports of decreased SorLA levels in late-onset AD patients (Scherzer et al., 2004) and polymorphisms in the SorLA gene are linked to both early and late-onset AD patients (Rogaeva et al., 2007; Grear et al., 2009; Caglayan et al., 2012; Pottier et al., 2012). These studies suggest that increasing the association between SorLA and APP by inhibiting the α_2A_ receptor could be beneficial for many AD patients. In APP/PS1 mice, a clinically used α_2_ receptor antagonist, idazoxan, ameliorates AD-related cognitive deficits in both novel object recognition and Morris water maze tests (Chen et al., 2014). Consistently, fluparoxan, another α_2_ receptor antagonist, improves the spatial working memory in a contextual fear conditioning task in these mice (Scullion et al., 2011). In humans, increased α_2A_ receptor density and/or activity have been associated with type 2 diabetes mellitus and depression (Cottingham et al., 2011; Cottingham and Wang, 2012), both of which are risk factors for AD. Under these disease conditions, the increase in the α_2A_ receptor may lead to α_2A_ receptor-promoted Aβ generation, which may act as a key contributor driving AD-related pathophysiology. Elevated α_2_ receptor density and response have also been reported in living AD patients. One study has found an increase in the α_2_ receptor density in platelets (Adunsky et al., 1989), and a few have found that both AD patients and aged individuals show increased norepinephrine levels in the CSF following stimulation or inhibition of the α_2_ receptors (Peskind et al., 1995; Raskind et al., 1999). Consistent with a role of the α_2A_ receptor promoting AD, our biostatistical analysis of the National Alzheimer's Coordinating Center database indicates that chronic activation of the α_2A_ receptor exacerbates disease progression in AD patients. Therefore, the α_2A_ receptor represents a previously unappreciated therapeutic target for AD.

A role of β receptors in AD pathogenesis and progression has also been suggested by pharmacological studies. The β_1_ blocker nevibolol reduces Aβ production in TG2576 mice with established impairment, even though it does not improve cognition function (Wang et al., 2013b). The β_2_ antagonist ICI 118,551 decreases Aβ load, while the β_2_ agonists isoproterenol and clenbuterol increase it, in APP/PS1 mice (Ni et al., 2006). Activation of the β_2_ receptor leads to increased trafficking of γ secretase to the late endosomes and lysosomes due to the association between β_2_ receptor and presenilin-1. The production of Aβ in these organelles is subsequently increased. Blockade of β_2_ receptors with ICI 118,551 also reduces Aβ generation associated with stress in non-transgenic C57 mice (Yu et al., 2010). However, in a 3xTg model of AD, ICI 118,551 increases Aβ levels and Aβ plaque burden as well as exacerbates cognitive deficits (Branca et al., 2014). The molecular mechanisms underlying the discrepancy of ICI 118,551 effects in different animal models remains to be investigated. Another β blocker, propranolol, which blocks β_1_ and β_2_ receptors with equal affinity (Summers, 2006), has been shown to lower BACE1 expression, decrease Aβ_42_ levels, decrease tau hyperphosphorylation, and improve cognitive impairments in both a mouse model of age-related cognitive decline (SAMP8) or corticosterone-treated mice (Dobarro et al., 2013a,b).

The β_2_ receptor can also mediate the effect of Aβ on tau phosphorylation (Wang et al., 2013a). In APP/PS1 mice, Aβ binds to and activates the β_2_ receptors, which leads to an increase in the activities of PKA and JNK. The β_2_-PKA-JNK pathway results in hyperphosphorylation of tau at specific loci. While the previous studies demonstrate that β agonists promote several aspects of AD pathology, there is also evidence that β agonists reduce, rather than promote, Aβ-related toxicity on neural plasticity and cognition. A β_2_ agonist, terbutaline, prevents the Aβ evoked inhibition of LTP (Wang et al., 2009b), and a β_3_ agonist, CL 316243, rescues Aβ induced memory loss in chicks (Gibbs et al., 2010). Overall, β receptors appear to execute diverse functions in multiple aspects related to AD pathology ranging from Aβ metabolism to toxicity, and further studies are needed to determine the role that each receptor subtype plays in the mechanism of AD pathogenesis.

Human genetic studies have indicated association of the β receptors with AD. A polymorphism in the β2 receptor contributes to AD onset in a Chinese group (Yu et al., 2008). Also, a combination of two single-nucleotide polymorphisms, a T allele in the G-protein β3 subunit (*GNB3*) and a C allele in the β2 adrenergic receptor (*ADRB1*), is associated with AD susceptibility. This is likely through enhanced cAMP/PKA signaling, which leads to an increase in APP expression (Bullido et al., 2004). Furthermore, hypertension patients who were taking beta blockers showed lower dementia incidence and cognitive decline rate (Khachaturian et al., 2006; Rosenberg et al., 2008), which suggests that blocking β receptors has a beneficial effect on AD.

## Summary and perspectives

The role of the noradrenergic system in cognition has long been known. Cognitive deficits occur after depletion of norepinephrine and improvements in cognitive function occur after increasing the availability of norepinephrine. Norepinephrine elicits its effects through the nine adrenergic receptors to which it binds. While the subtype-specific role of each adrenergic receptor in cognition remains to be elucidated, in general, α_1_ and β receptors are considered stimulatory, enhancing neurotransmission and plasticity. On the other hand, α_2_ receptors are inhibitory, reducing NE release and neuronal excitability. In addition to the noradrenergic system being important in cognitive function in normal circumstances, many changes occur in the noradrenergic system in AD patients. The degeneration of noradrenergic neurons in the locus coeruleus of AD patients was an observation made over 30 years ago, and more recent research has found changes that occur within the remaining neurons, likely as compensatory mechanisms. This neuronal loss and the resultant compensatory mechanisms lead to changes in the level of norepinephrine available in the brain, which consequently affect cognitive functions. Evidence from both animal and human studies suggest that loss of noradrenergic input significantly exacerbates AD-related cognitive deficits.

Familial AD with mutations in genes encoding APP or PS1 accounts for less than 10% of AD. In contrast, late-onset sporadic AD likely involves multiple genetic and environmental risk factors that lead to disruption of amyloid homeostasis. The pivotal role that the noradrenergic system plays in supporting interactions with and responses to environmental stimuli, as well as alterations of this system in the early stage of AD, suggest a potential contribution of noradrenergic dysfunction to AD pathogenesis. Supporting this notion, activation of α_2A_ and β_2_ receptors directly regulates Aβ generation. More studies are needed to address the potential role of each adrenergic receptor subtype in amyloid metabolism and tau pathogenesis. The available receptor knockout mice for each subtype (Philipp and Hein, 2004) provide useful tools to address this. Genetic studies could also be done to look for any polymorphisms within the adrenergic receptor genes that are associated with late-onset AD.

Despite the fact that a clear picture of the roles of various noradrenergic components in AD pathogenesis is still lacking, current evidence points to the α_2A_ receptors as a novel target for AD. This receptor subtype is the key regulator of the noradrenergic activity, inhibiting both noradrenergic input to the cerebral cortex and the resulting response in this brain region (Hein, 2006). In addition, our recent studies have demonstrated that activation of the α_2_ receptor promotes amyloidogenic processing of the endogenous APP and amyloid-related pathology (Chen et al., 2014). Therefore, a pharmacological blockade of α_2A_ receptors can both increase NE release, which enhances NE-dependent cognitive and neuroprotective effects, and decrease Aβ generation, which reduces Aβ-dependent toxicity. Increased α_2A_ receptor density and/or activity have been observed in living AD patients and in patients at high risk for AD, such as those with type 2 diabetes mellitus and depression. Blockade of α_2A_ receptors would be particularly effective in patients with these receptors upregulated.

While a novel target for AD, adrenergic receptors are an established target for other disorders including cardiovascular, behavioral, and mood disorders. Because of this, extensive drug discovery research has already been done and a number of noradrenergic receptor drugs, including α_2_ receptor blockers, already exist and are FDA approved. Repurposing these drugs could greatly expedite the timeframe for getting a novel AD drug on the market. In fact, some noradrenergic drugs have been tested in AD patients to alleviate some of the mood symptoms associated with AD. For example, tricyclic antidepressants, which block norepinephrine transporters, have been used to treat depressed AD patients (Sallee and Pollock, 1990; Teri et al., 1991), and both α_1_ and β receptor antagonists can improve agitation in AD (Pauszek, 1991; Shankle et al., 1995; Peskind et al., 2005; Wang et al., 2009a). While these noradrenergic drugs are effective in the treatment of the behavioral and mood disorders associated with AD, further research is needed to elucidate their effect on cognition. Once this is known, it will be possible to quickly repurpose these drugs in order to treat the cognitive aspects of AD.

## Author contributions

MG, PC, YC, KJ, and QW planned the structure of the manuscript. MG, PC, and YC prepared the manuscript. MG, KJ, ER, and QW edited and restructured the manuscript.

### Conflict of interest statement

The authors declare that the research was conducted in the absence of any commercial or financial relationships that could be construed as a potential conflict of interest.
